# A precise estimation for vibrational energies of diatomic molecules using the improved Rosen–Morse potential

**DOI:** 10.1038/s41598-023-37888-2

**Published:** 2023-07-18

**Authors:** M. Abu-Shady, E. M. Khokha

**Affiliations:** 1 Department of Mathematics and Computer Science, Faculty of Science, Menoufia University, Shibin El Kom, 32511 Menoufia Egypt; 2 Faculty of Computer Science and Engineering, King Salman International University (KSIU), El Tor 46511, South Sinai, Egypt

**Keywords:** Chemistry, Mathematics and computing, Physics

## Abstract

In the context of the generalized fractional derivative, novel solutions to the *D*-dimensional Schrödinger equation are investigated via the improved Rosen-Morse potential (IRMP). By applying the Pekeris-type approximation to the centrifugal term, the generalized fractional Nikiforov-Uvarov method has been used to derive the analytical formulations of the energy eigenvalues and wave functions in terms of the fractional parameters in *D*-dimensions. The resulting solutions are employed for a variety of diatomic molecules (DMs), which have numerous uses in many fields of physics. With the use of molecular parameters, the IRMP is utilized to reproduce potential energy curves for numerous DMs. The pure vibrational energy spectra for several DMs are determined using both the fractional and the ordinary forms to demonstrate the effectiveness of the method utilized in this work. As compared to earlier investigations, it has been found that our estimated vibrational energies correspond with the observed Rydberg-Klein-Rees (RKR) data much more closely. Moreover, it is observed that the vibrational energy spectra of different DMs computed in the existence of fractional parameters are superior to those computed in the ordinary case for fitting the observed RKR data. Thus, it may be inferred that fractional order significantly affects the vibrational energy levels of DMs. Both the mean absolute percentage deviation (MAPD) and average absolute deviation (AAD) are evaluated as the goodness of fit indicators. According to the estimated AAD and MAPD outcomes, the IRMP is an appropriate model for simulating the RKR data for all of the DMs under investigation.

## Introduction

In recent decades, numerous works on the solutions to the Klein-Gordon, Dirac and Schrödinger equations were reported^1–6^. This is owing to the reality that the solutions to these wave equations include all of the data required for the quantum system under investigation. In this context, the vibrational energy spectra of diatomic molecules (DMs) were investigated using several potential functions, such as the Morse^7^, Kratzer^8^, Deng-Fan^9^, Hulthén^10^, Tietz-Hua^11^ and others.

In 1932, Rosen and Morse^12^ suggested a diatomic molecular function

1 $$\begin{aligned} U(r)=C\,\text {tanh}\big (r/d)-B\,\text {sech}^2\big (r/d), \end{aligned}$$

where *C*, *B* and *d* are changeable parameters. The Rosen-Morse potential (RMP) was used to explore polyatomic vibrational states of the NH${_3}$ molecule^12^. It was also employed to characterize the diatomic molecular vibrations^13^. By utilizing the equilibrium bond length (${r_e}$) and the dissociation energy (${D_e}$) for a DM as explicit parameters, Jia et al.^14^ presented an improved expression of the RMP based on the original form of the RMP.

2 $$\begin{aligned} V(r)=D_e\Big (1-\frac{e^{\alpha r_e}+1}{e^{\alpha r}+1}\Big )^2, \end{aligned}$$

where the screening parameter ${\alpha }$ is defined as follow^15^:

3 $$\begin{aligned} \alpha =\sqrt{\frac{k_e}{2D_e}}+\frac{1}{r_e}W\Bigg (r_e\sqrt{\frac{k_e}{2D_e}}e^{-r_e\sqrt{\frac{k_e}{2D_e}}}\Bigg ), \qquad k_e=4\mu \pi ^2 c^2 w_e^2 \end{aligned},$$

where ${w_e}$ is the equilibrium harmonic vibrational frequency and *W* is the Lambert *W* function^16^ that fulfils ${z=W(z)e^{W(z)}}$. The improved Rosen-Morse potential (IRMP) was extensively employed to depict the diatomic molecular vibrations by solving the relativistic and non-relativistic wave equations.

Wang et al.^17^ demonstrated that for diatomic molecules, one form of the Schiöberg potential is identical to the IRMP. Chen et al.^18^ used the supersymmetric shape invariance method to find the solutions of the Klein-Gordon equation (KGE) with the IRMP and determined the relativistic vibrational transition frequencies for the ${3^3\Sigma _g^+}$ state of the Cs${}_{2}$ molecule. The ro-vibrational energy levels for the ${5^1\Delta _g^+}$ state of the Na${}_{2}$ molecule and the ${3^3\Sigma _g^+}$ state of the Cs${}_{2}$ molecule were calculated with the IRMP in *D*-dimensions using different techniques^19–21^. By using the parametric Nikiforov‑Uvarov (NU) method, Akanni and Kazeem^22^ derived the approximate solutions of the KGE with the IRMP. The thermodynamics properties for the Na$_{2}$ dimer were discussed using the IRMP^23^. The authors in Ref.^15^ examined the solutions of the Dirac equation with the IRMP and computed the relativistic vibrational energy spectra for the ${3^3\Sigma _g^+}$ state of the Cs${}_{2}$ molecule.

Based on the IRMP, the predictions of molar enthalpy, entropy and Gibbs free energy for the P${}_{2}$ dimer were calculated^24–26^. Udoh et al.^27^ utilized the NU method to find the solutions of the Schrödinger equation (SE) in *D*-dimensions for the IRMP and estimated the ro-vibrational energies of H${}_{2}$(${X^1\Sigma _g^+}$) and NO(${a^4\Pi _i}$) diatomic molecules. Horchani and Jelassi^28^ used the IRMP to explore the impact of quantum correction on the thermodynamic characteristics of the Cs${}_{2}$ (${3^3\Sigma _g^+}$) molecule. The vibrational energies for nitrogen molecule and sodium dimer were found^29^ by studying the solutions of the SE with the IRMP. Al-Raeei^30^ derived an expression of the bond equilibrium length of the IRMP and used it to analyze six dimers and molecules.Yanar^31^ computed the vibrational energies of the SiF${^+}$(${X^1\Sigma ^+}$) molecule utilizing particular cases of the general molecular potential, such as the Morse potential, IRMP, and others.

Fractional derivatives calculus has been an appealing area of research in recent decades because of its application in different scientific fields such as physics, chemistry, biology, engineering, medicine, and economics. In the literature, various fractional derivative definitions have been introduced, such as Riemann-Liouville^32^, Caputo^33^, Jumarie^34^, and others^35^.

According to Khalil^36^, an alternative fractional derivative definition that preserves classical features is the conformable fractional derivative (CFD). In the context of the CFD, the characteristics of heavy mesons were discussed using the *N*-dimensional radial SE for the trigonometric RMP^37^, hot-magnetized inter-action potential^38^, dependent temperature potential^39^, and generalized Cornell potential^40^. Abu-Shady^41^ used the concept of the CFD to present the mathematical model for describing the Coronavirus disease (COVID-19).

The generalized fractional derivative (GFD) is a novel concept for the fractional derivative that produces results consistent with those of classical definitions, was recently proposed by Abu-Shady and Kaabar^42^. The extended NU method was employed in conjunction with the GFD to solve the SE and determine the masses of heavy mesons^43^ and also the mass spectra of heavy tetraquarks and diquark^44^. The masses of heavy flavor baryons with and without hyperfine interactions were calculated using the generalized fractional iteration approach in Ref.^45^. In the scope of the GFD, the analytical exact iteration method was used to analyze the thermodynamic properties of heavy mesons in strongly coupled quark-gluon plasma^46^. In Ref.^47^, the fractional forms of various special functions were derived using the GFD. By using the generalized fractional Nikiforov‑Uvarov (GFNU) method^48^, the solutions of the SE with the generalized Woods-Saxon potential were derived. More recently, the *D*-dimensional SE was studied via the GFNU technique using the Deng-Fan potential^49^ and the improved Tietz potential (ITP)^50^. Furthermore the vibrational and ro-vibrational energies of several DMs were predicted.

It is vital to note that no previous research into SE solutions for the IRMP has been disclosed within the framework of the GFD. To this end, the purpose of this work is to explore solutions to the *D*-dimensional SE for the IRMP in the scope of the GFD. The structure of this work is as follows: The basics of the GFNU approach are explained in Section “The basics of the GFNU method”. The solutions of the *D*-dimensional SE for the IRMP are found within the scope of the GFD in Section “Solution of the SE with the IRMP in* D*-dimensions”. The numerical results of the vibrational energy levels of different DMs are provided and analyzed in Section “Discussion”. Finally, Section “Conclusion” provides a succinct conclusion of the work.

## The basics of the GFNU method

The basics of the GFNU method are introduced in this part for solving the generalized fractional differential equation, which takes the following form^49, 50^.

4 $$\begin{aligned} {D^\gamma [D^\gamma \mathcal {W}(z)]+\frac{\tilde{\tau }(z)}{\sigma (z)}D^\gamma \mathcal {W} (z)+\frac{\tilde{\sigma }(z)}{\sigma ^2(z)}\mathcal {W} (z)=0,} \end{aligned}$$

where ${\tilde{\sigma }(z)}$ and ${\sigma (z)}$ are polynomials of maximum ${2\gamma }$-th degree and ${\tilde{\tau }(z)}$ is a function at most ${\gamma }$-th degree. Utilizing the primary characteristics of the GFD^42^

5 $$\begin{aligned} D^\gamma \mathcal {W}(z)= & {} Qz^{1-\gamma }\mathcal {W}'(z), \end{aligned}$$

6 $$\begin{aligned} D^\gamma [D^\gamma \mathcal {W}(z)]= & {} Q^2\Big [z^{2(1-\gamma )}\mathcal {W}''(z)+(1-\gamma )z^{1-2\gamma }\mathcal {W}'(z)\Big ], \end{aligned}$$

where

7 $$\begin{aligned} Q=\frac{\Gamma (\beta )}{\Gamma (\beta -\gamma +1)}, \end{aligned}$$

with

8 $$\begin{aligned} 0<\gamma \leqslant 1, \qquad \beta \in R^+. \end{aligned}$$

and inserting Eqs. (5) and (6) into Eq. (4) gives

9 $$\begin{aligned} \mathcal {W}''(z)+\frac{Q(1-\gamma )z^{-\gamma }\sigma (z)+\tilde{\tau }(z)}{Qz^{1-\gamma }\sigma (z)}\mathcal {W}' (z)+\frac{\tilde{\sigma }(z)}{Q^2z^{2-2\gamma }\sigma ^2(z)}\mathcal {W}(z)=0, \end{aligned}$$

Eq. (4) can be changed into the hypergeometric equation shown below:

10 $$\begin{aligned} {\mathcal {W}''(z)+\frac{\tilde{\tau }_{GF}(z)}{\sigma _{GF}(z)}\mathcal {W}' (z)+\frac{\tilde{\sigma }(z)}{\sigma _{GF}^2(z)}\mathcal {W}(z)=0,} \end{aligned}$$

where

11 $$\begin{aligned} \tilde{\tau }_{GF}(z)=Q(1-\gamma )z^{-\gamma }\sigma (z)+\tilde{\tau }(z), \qquad \sigma _{GF}(z)=Qz^{1-\gamma }\sigma (z). \end{aligned}$$

where the generalized fractional is denoted by the subscript GF. Now taking

12 $$\begin{aligned} \mathcal {W} (z)= X(z) Y(z). \end{aligned}$$

and putting Eq. (12) into Eq. (10) leads to

13 $$\begin{aligned} \sigma _{GF}(z) Y''(z)+\tau _{GF}(z) Y'(z)+g(z) Y(z)=0. \end{aligned}$$

where *X*(*z*) is given by:

14 $$\begin{aligned} X(z)=\text {exp}\Big (\int \frac{\pi _{GF} (z)}{\sigma _{GF} (z)} \, dz\Big ). \end{aligned}$$

and

15 $$\begin{aligned} g(z)=h(z)+\pi _{GF}'(z). \end{aligned}$$

The function ${Y(z)}=Y_\nu (z)$ is a hypergeometric-type function with polynomial solutions provided by the Rodrigues formula

16 $$\begin{aligned} Y_\nu (z)=\frac{C_\nu }{\rho (z)}\frac{d^\nu }{dz^\nu }[\sigma _{GF}^\nu (z) \rho (z)], \end{aligned}$$

where ${C_\nu }$ is a constant of the normalization, and ${\rho (z)}$ is the weight function given by:

17 $$\begin{aligned} \rho (z)=\Big [\sigma _{GF}(z)\Big ]^{-1}\text {exp}\Big (\int \frac{\tau _{GF} (z)}{\sigma _{GF} (z)} \, dz\Big ). \end{aligned}$$

The polynomial ${\pi _{GF}(z)}$ is determined by:

18 $$\begin{aligned} \pi _{GF}(z)=\frac{\sigma _{GF}'(z)-\tilde{\tau }_{GF}(z)}{2}\pm \sqrt{\Bigg [\frac{\sigma _{GF}'(z)-\tilde{\tau }_{GF}(z)}{2}\Bigg ]^2-\tilde{\sigma }(z)+h(z)\sigma _{GF}(z)}, \end{aligned}$$

The function *h*(*z*) can be obtained if the function under the square root is the square of a polynomial. Hence, the eigenvalue expression is:

19 $$\begin{aligned} g(z)=g_\nu (z)=-\nu \Big [\tau _{GF}'(z)+\frac{(\nu -1)}{2}\sigma _{GF}''(z)\Big ], \end{aligned}$$

where

20 $$\begin{aligned} \tau _{GF}(z)=\tilde{\tau }_{GF}(z)+2\pi _{GF}(z). \end{aligned}$$

Finally, by putting Eqs. (14) and (16) into Eq. (12), the eigenfunctions ${\mathcal {W}(z)}$ can be determined.

## Solution of the SE with the IRMP in *D*-dimensions

The radial SE for a DM in the *D*-dimensional space with the potential *V*(*r*) is given by^50^.

21 $$\begin{aligned} \Biggl \{\frac{d^2}{dr^2}+\frac{D-1}{r}\frac{d}{dr}-\frac{J(J+D-2)}{r^2}+\frac{2\mu }{\hbar ^2}\Big (E-V(r)\Big )\Biggl \}G(r)=0, \end{aligned}$$

where *E*, *D*, *J* and are the energy eigenvalue, the dimensionality number, and the vibrational quantum number respectively, and ${\hbar }$ is the reduced Planck’s constant. By putting,

22 $$\begin{aligned} G(r)=r^\frac{1-D}{2} \mathcal {H}(r). \end{aligned}$$

Eq. (21) turns to

23 $$\begin{aligned} \frac{d^2\mathcal {H}(r)}{dr^2}+\Bigg [\frac{2\mu }{\hbar ^2}\Big (E-V(r)\Big )-\frac{(\delta ^2-\frac{1}{4})}{r^2}\Bigg ]\mathcal {H}(r)=0, \end{aligned}$$

with

24 $$\begin{aligned} \delta =J+\frac{D-2}{2}. \end{aligned}$$

Inserting the IRMP (2) into Eq. (23) gives:

25 $$\begin{aligned} \frac{d^2\mathcal {H}(r)}{dr^2}+\Biggl \{\frac{2\mu }{\hbar ^2}\Bigg [E-D_e\Big (1-\frac{e^{\alpha r_e}+1}{e^{\alpha r}+1}\Big )^2\Bigg ]-\frac{(\delta ^2-\frac{1}{4})}{r^2}\Bigg )\Biggl \}\mathcal {H}(r)=0. \end{aligned}$$

To determine the approximate analytical solutions of Eq. (25), the Pekeris approximation recipe is applied to the centrifugal term ${(\delta ^2-\frac{1}{4})\big /r^2}$ as^19–21^

26 $$\begin{aligned} \frac{\delta ^2-\frac{1}{4}}{r^2}\approx \frac{\delta ^2-\frac{1}{4}}{r_e^2}\Bigg [b_0+\frac{b_1}{(e^{\alpha r}+1)}+\frac{b_2}{(e^{\alpha r}+1)^2}\Bigg ]. \end{aligned}$$

where the coefficients ${b_0, b_1}$ and ${b_2}$ are defined as follows^19–21^

27 $$\begin{aligned} b_0= & {} 1+\frac{1}{\alpha ^2 r_e^2}\Bigg [3-3\alpha r_e+6 e^{-\alpha r_e}+3 e^{-2\alpha r_e}-2\alpha r_e e^{-\alpha r_e}+\alpha r_ee^{-2\alpha r_e}\Bigg ], \end{aligned}$$

28 $$\begin{aligned} b_1= & {} \frac{2}{\alpha ^2 r_e^2}\Bigg [-9+3\alpha r_e-3e^{\alpha r_e}+2\alpha r_e e^{\alpha r_e}-9 e^{-\alpha r_e}-3e^{-2\alpha r_e}-\alpha r_ee^{-2\alpha r_e}\Bigg ], \end{aligned}$$

29 $$\begin{aligned} b_2= & {} \frac{1}{\alpha ^2 r_e^2}\Bigg [18+12 e^{\alpha r_e}+3e^{2\alpha r_e}-2\alpha r_e e^{\alpha r_e}-\alpha r_ee^{2\alpha r_e}+12 e^{-\alpha r_e}+3e^{-2\alpha r_e}+2\alpha r_ee^{-\alpha r_e}+\alpha r_ee^{-2\alpha r_e}\Bigg ]. \end{aligned}$$

Inserting Eq. (26) into Eq. (25) yields

30 $$\begin{aligned} \frac{d^2H(r)}{dr^2}+\Biggl \{\frac{2\mu }{\hbar ^2}\Bigg [E-D_e\Big (1-\frac{e^{\alpha r_e}+1}{e^{\alpha r}+1}\Big )^2\Bigg ]-\frac{\delta ^2-\frac{1}{4}}{r_e^2}\Bigg [b_0+\frac{b_1}{(e^{\alpha r}+1)}+\frac{b_2}{(e^{\alpha r}+1)^2}\Bigg ]\Biggl \}H(r)=0, \end{aligned}$$

By employing the variable ${z=-e^{-\alpha r}}$, Eq. (30) turns into

31 $$\begin{aligned} H''(z)+\frac{(1-z)}{z(1-z)}H'(z)+\frac{1}{z^2(1- z)^2}\Big [-A_1z^2+A_2z-A_3\Big ]H(z)=0, \end{aligned}$$

where

32 $$\begin{aligned} A_1= & {} \eta \big (b_0+b_1+b_2\big )+\xi e^{-2\alpha r_e}-\epsilon , \end{aligned}$$

33 $$\begin{aligned} A_2= & {} \eta \big (2b_0+b_1\big )-2\xi e^{-\alpha r_e}-2\epsilon , \end{aligned}$$

34 $$\begin{aligned} A_3= & {} \eta b_0+\xi -\epsilon , \end{aligned}$$

with

35 $$\begin{aligned} \eta =\frac{\delta ^2-\frac{1}{4}}{\alpha ^2r_e^2}, \qquad \xi =\frac{2\mu D_e}{\alpha ^2\hbar ^2}, \qquad \epsilon =\frac{2\mu E}{\alpha ^2\hbar ^2}. \end{aligned}$$

By changing the integer orders in Eq. (31) to fractional orders, the generalized fractional version of the SE for the IRMP is being represented as follows:

36 $$\begin{aligned} D^\gamma \big [D^\gamma H(z)\big ]+\frac{(1- z^\gamma )}{z^\gamma (1- z^\gamma )}D^\gamma \big [H(z)\big ]+\frac{1}{z^{2\gamma }(1- z^\gamma )^2}\Big [-A_1z^{2\gamma }+A_2z^\gamma -A_3\Big ]H(z)=0, \end{aligned}$$

Inserting Eqs. (5) and (6) into Eq. (36) yields

37 $$\begin{aligned} H''(z)+\frac{\Big [Q(1-\gamma )+1\Big ](1- z^\gamma )}{Qz(1- z^\gamma )}H'(z)+\frac{1}{Q^2z^2(1- z^\gamma )^2}\Big [-A_1z^{2\gamma }+A_2z^\gamma -A_3\Big ]H(z)=0, \end{aligned}$$

By comparing Eq. (37) with Eq. (10) yields the following functions:

38 $$\begin{aligned} \tilde{\tau }_{GF}(z)=\Big (Q(1-\gamma )+1\Big )(1-z^\gamma ), \qquad \sigma _{GF}(z)=Qz(1-z^\gamma ), \qquad \tilde{\sigma }_{GF}(z)=-A_1z^{2\gamma }+A_2z^\gamma -A_3. \end{aligned}$$

By putting Eq. (38) into Eq. (18), the function ${\pi _{GF}(z)}$ is found as follows:

39 $$\begin{aligned} \begin{aligned}{}&\pi _{GF}(z)=\frac{(Q\gamma -1)+(1-2Q\gamma )z^\gamma }{2}\pm \\ {}&\sqrt{\Big [\frac{(1-2Q\gamma )^2}{4}+A_1- Qhz^{1-\gamma }\Big ]z^{2\gamma }+\Big [\frac{(Q\gamma -1)(1-2Q\gamma )}{2}-A_2+Qhz^{1-\gamma }\Big ]z^\gamma +\Big [\frac{(Q\gamma -1)^2}{4}+A_3\Big ]}. \end{aligned} \end{aligned}$$

Eq. (39) can be reduced to the following:

40 $$\begin{aligned} \pi _{GF}(z)=\frac{(Q\gamma -1)+(1-2Q\gamma )z^\gamma }{2}\pm \sqrt{T_1 z^{2\gamma }+T_2z^\gamma +T_3}, \end{aligned}$$

where

41 $$\begin{aligned} T_1=B_1-Qhz^{1-\gamma }, \qquad T_2=B_2+Qhz^{1-\gamma }, \qquad T_3=B_3, \end{aligned}$$

with

42 $$\begin{aligned} B_1=\frac{(1-2Q\gamma )^2}{4}+A_1, \qquad B_2=\frac{(Q\gamma -1)(1-2Q\gamma )}{2}-A_2, \qquad B_3=\frac{(Q\gamma -1)^2}{4}+A_3. \end{aligned}$$

By applying the restriction that the discriminant of the function under the square root of Eq. (40) should be zero, the function *h*(*z*) can be found as follow

43 $$\begin{aligned} h_\pm =\lambda \Biggl [-\big (B_2+2 B_3\big )\pm 2\sqrt{B_3\Big (B_1+B_2+B_3\Big )}\Biggl ]z^{\gamma -1}; \qquad \lambda =\frac{1}{Q}. \end{aligned}$$

By inserting Eq. (43) into Eq. (40) yields

44 $$\begin{aligned} \pi _{GF}(z)=\frac{(Q\gamma -1)+(1-2Q\gamma )z^\gamma }{2}\pm {\left\{ \begin{array}{ll} \Big (\sqrt{B_3}-\sqrt{B_1+B_2+B_3}\Big )z^\gamma -\sqrt{B_3}, \qquad h=h_+ \\ \Big (\sqrt{B_3}+\sqrt{B_1+B_2+B_3}\Big )z^\gamma -\sqrt{B_3}, \qquad h=h_- \\ \end{array}\right. }. \end{aligned}$$

The negative sign in Eq. (44) is selected to get a physically acceptable solution, the ${\pi _{GF}(z)}$ then changes to

45 $$\begin{aligned} \pi _{GF}(z)=\frac{(Q\gamma -1)+(1-2Q\gamma )z^\gamma }{2}-\Big (\sqrt{B_3}-\sqrt{B_1+B_2+B_3}\Big )z^\gamma +\sqrt{B_3}, \end{aligned}$$

and

46 $$\begin{aligned} h=\lambda \Biggl [-\big (B_2+2B_3\big )+2\sqrt{B_3\Big (B_1+B_2+B_3\Big )}\Biggl ]z^{\gamma -1}. \end{aligned}$$

Therefore, the functions ${g(z), \tau _{GF}(z)}$ and ${g_\nu (z)}$ are written as follows:

47 $$\begin{aligned} g(z)= & {} \Bigg [-\lambda \big (B_2+2B_3\big )-\sqrt{B_3}\Big ( \gamma -2\lambda \sqrt{B_1+B_2+B_3}\Big )+\gamma \Bigg (\frac{1}{2}\Big (1-2Q\gamma \Big )+\sqrt{B_1+B_2+B_3}\Bigg )\Bigg ]z^{\gamma -1}, \end{aligned}$$

48 $$\begin{aligned} \tau _{GF}(z)= & {} \Big (2\sqrt{B_3}+Q\Big )-\Big [Q(\gamma +1)+2\Big (\sqrt{B_3}-\sqrt{B_1+B_2+B_3}\Big )\Big ]z^\gamma , \end{aligned}$$

49 $$\begin{aligned} g_\nu (z)= & {} \nu \gamma \Big [\frac{Q(\nu +1)(\gamma +1)}{2}+2\Big (\sqrt{B_3}-\sqrt{B_1+B_2+B_3}\Big )\Big ]z^{\gamma -1}. \end{aligned}$$

By integrating Eqs. (47) and (49), the fractional form of the energy eigenvalue of a DM in *D* dimensions can be expressed as:

50 $$\begin{aligned} \begin{aligned} E_{Frac.}=&\frac{\alpha ^2\hbar ^2}{2\mu }\Bigg [V_3+\frac{(Q\gamma -1)^2}{4}\Bigg ]\\ {}&-\frac{\alpha ^2\hbar ^2}{2\mu }\Bigg [\frac{\gamma \Big (\omega -(2\nu +1)\sqrt{\frac{Q^2\gamma ^2}{4}+V_3- V_2+V_1}\Big )+\lambda \Big (\frac{Q\gamma (1-Q\gamma )}{2}+2 V_3-V_2\Big )}{2\lambda \sqrt{\frac{Q^2\gamma ^2}{4}+V_3- V_2+V_1}-\gamma (2\nu +1)}\Bigg ]^2, \end{aligned} \end{aligned}$$

where

51 $$\begin{aligned} \omega= & {} \frac{1}{2}\Big [Q\nu (\nu +1)(\gamma +1)+2Q\gamma -1\Big ], \qquad V_1=\gamma \big (b_2+b_1+b_0\big )+\xi e^{-2\alpha r_e}, \end{aligned}$$

52 $$\begin{aligned} V_2= & {} \eta \big (b_1+2b_0\big )+2\xi e^{-\alpha r_e}, \qquad V_3=\eta b_0+\xi . \end{aligned}$$

In the absence of the influence of the fractional parameters, the following ordinary expression for the energy eigenvalues can be produced by putting ${\gamma =\beta =1}$:

53 $$\begin{aligned} E_{Ord.}=\frac{\alpha ^2\hbar ^2}{2\mu }\Biggl \{V_3-\frac{\big [\nu (\nu +1)+\frac{1}{2}\big ]-(2\nu +1)\sqrt{V_3- V_2+V_1+\frac{1}{4}}+2 V_3-V_2}{\sqrt{V_3-V_2+V_1+\frac{1}{4}}-(2\nu +1)}\Biggl \}^2. \end{aligned}$$

By utilizing Eq. (14), the function *X*(*z*) becomes

54 $$\begin{aligned} X(z)=z^{\lambda \Big (\frac{(Q\gamma -1)}{2}+\sqrt{B_3}\Big )}\Big (1- z^\gamma \Big )^{\Big ({\frac{1}{2}-\frac{\lambda }{\gamma }\sqrt{B_1+B_2+B_3}}\Big )}. \end{aligned}$$

Using Eq. (17), the function ${\rho (z)}$ can be stated as follows

55 $$\begin{aligned} \rho (z)=\lambda z^{2\lambda \sqrt{B_3}}\Big (1- z^\gamma \Big )^{{-\frac{2\lambda }{\gamma }\sqrt{B_1+B_2+B_3}}}. \end{aligned}$$

With the help of Eq. (16), the function ${Y_\nu (z)}$ is written as

56 $$\begin{aligned} Y_\nu (z) =C_\nu z^{-2\lambda \sqrt{B_3}}\Big (1- z^\gamma \Big )^{{\frac{2\lambda }{ \gamma }\sqrt{B_1+B_2+B_3}}}\frac{d^\nu }{dz^\nu }\Bigg [Q^\nu z^{\Big (\nu +2\lambda \sqrt{B_3}\Big )}\Big (1- z^\gamma \Big )^{{\Big (\nu -\frac{2\lambda }{ \gamma }\sqrt{B_1+B_2+B_3}\Big )}}\Bigg ]. \end{aligned}$$

The complete solution of Eq. (31) is obtained by applying Eq. (12) as follows

57 $$\begin{aligned} H(z)=C_\nu z^{\lambda \Big (\frac{(Q\gamma -1)}{2}-\sqrt{B_3}\Big )}\Big (1-z^\gamma \Big )^{\Big ({\frac{1}{2}+\frac{\lambda }{ \gamma }\sqrt{B_1+B_2+B_3}}\Big )}\frac{d^\nu }{dz^\nu }\Bigg [Q^\nu z^{\Big (\nu +2\lambda \sqrt{B_3}\Big )}\Big (1- z^\gamma \Big )^{{\Big (\nu -\frac{2\lambda }{ \gamma }\sqrt{B_1+B_2+B_3}\Big )}}\Bigg ]. \end{aligned}$$

**Table 1:** Molecular parameters of the chosen DMs.

Molecule	State	${r_e}$ (${\overset{o}{A}}$)	${D_e}$(eV)	${\omega _e}$ ${(cm^{-1})}$	${\mu }$ (a.m.u.)	Ref.
ScI	$B^1\prod$	2.7215	4.098	243.43	33.1961	^51^
$\text {N}_2$	$X^1\Sigma _g^+$	1.0977	9.904	2358.57	7.00335	^52^
$\text {K}_2$	$X^1\Sigma _g^+$	3.9253	0.742	92.4054	19.4818	^53^
$\text {SiF}^+$	$X^1\Sigma ^+$	1.5265	6.600	1050.37	11.3320	^54^
SiN	$X^2\Sigma ^+$	1.5700	4.595	1151.36	9.34588	^55^
SiP	$X^2\prod$	2.0775	3.768	615.70	14.7294	^56^
SrO	$X^1\Sigma ^+$	1.9198	4.610	653.49	13.5287	^57^
ScO	$X^2\Sigma ^+$	1.6682	6.759	964.90	11.7997	^58^
AsP	$X^1\Sigma ^+$	1.9990	4.610	567.94	21.9141	^59^
AsS	$X^2\prod$	2.0174	4.280	604.02	22.4523	^60^
CS	${X^1\Sigma ^+}$	1.5349	7.354	1285.08	8.72520	^61^
YO	$X^2\Sigma ^+$	1.7900	7.289	861.00	13.5590	^58^

**Figure 1:**
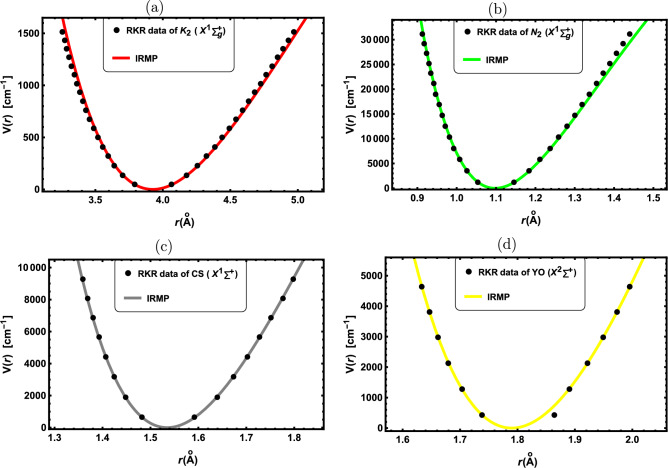
RKR data points and IRMP for the: (**a**) K${}_{2}$(${X^1\Sigma _g^+}$), (**b**) N${}_{2}$(${X^1\Sigma _g^+}$), (**c**) CS(${X^1\Sigma ^+}$) and (d) YO(${X^2\Sigma ^+}$).

**Figure 2:**
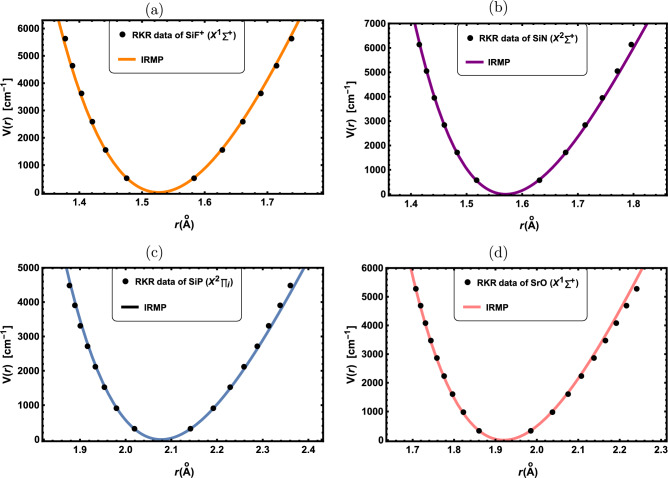
RKR data points and IRMP for the: (**a**) SiF${^+}$(${X^1\Sigma ^+}$), (**b**) SiN(${X^2\Sigma ^+}$), (**c**) SiP(${X^2\prod }$) and (**d**) SrO(${X^1\Sigma ^+}$).

**Figure 3:**
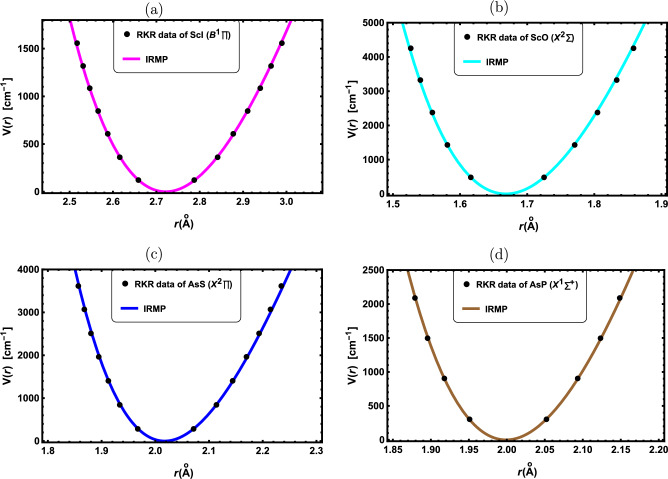
RKR data points and IRMP for the: (**a**) ScI(${B^1\prod }$), (**b**) ScO(${X^2\Sigma ^+}$), (**c**) AsS(${X^2\prod }$) and (**d**) AsP(${X^1\Sigma ^+}$).

**Table 2:** Estimated AAD for the IRMP.

Molecule	ScI(${B^1\prod }$)	N${}_{2}$(${X^1\Sigma _g^+}$)	K${}_{2}$(${X^1\Sigma _g^+}$)	SiF${^+}$(${X^1\Sigma ^+}$)	SiN(${X^2\Sigma ^+}$)	SiP(${X^2\prod }$)
AAD${\%}$	0.0357	0.6340	0.6999	0.1656	0.1939	0.2715

Molecule	SrO(${X^1\Sigma ^+}$)	ScO(${X^2\Sigma ^+}$)	AsS(${X^2\prod }$)	AsP(${X^1\Sigma ^+}$)	CS(${X^1\Sigma ^+}$)	YO(${X^2\Sigma ^+}$)
AAD${\%}$	0.2842	0.0907	0.0411	0.0969	0.0627	0.0470

**Table 3:** Calculated energies (${cm^{-1}}$) for ScI (${B^1\prod }$) molecule (${\gamma =0.8598, \ \beta =0.6859}$).

${\nu }$	RKR^51^	Ref.^63^	Ref.^64^	Ref.^65^	Eq. (53)	Eq. (50)
0	121.5	121.567	121.569	121.6	121.692	121.650
1	363.7	363.712	363.695	363.7	363.889	363.760
2	604.6	604.572	604.484	604.6	604.851	604.630
3	844.3	844.147	843.938	844.1	844.575	844.261
4	1082.7	1082.437	1082.059	1082.4	1083.063	1082.650
5	1319.8	1319.441	1318.846	1319.4	1320.313	1319.799
6	1555.7	1555.159	1554.301	1555.2	1556.323	1555.705
MAPD%		0.0239	0.0488	0.0280	0.0566	0.0221

**Table 4:** Calculated energies (${cm^{-1}}$) for N${}_{2}$(${X^1\Sigma _g^+}$) molecule (${\gamma =0.9585, \ \beta =0.6842}$).

${\nu }$	RKR^52^	Ref.^66^	Morse^52^	DMRM^52^	Eq. (53)	Eq. (50)
0	1184.454	1175.820	1174.933	1174.997	1174.935	1183.966
1	3526.358	3502.697	3498.685	3499.841	3500.195	3526.869
2	5833.452	5794.661	5787.619	5790.876	5792.0565	835.821
3	8107.046	8051.712	8041.735	8048.081	8050.455	8110.759
4	10348.312	10273.850	10261.034	10271.387	10275.326	10351.617
5	12558.287	12461.076	12445.514	12460.752	12466.605	12558.331
6	14737.876	14613.389	14595.177	14616.138	14624.226	14730.836
7	16887.860	16730.789	16710.022	16737.473	16748.124	16869.065
8	19008.894	18813.278	18790.048	18824.747	18838.234	18972.953
9	21101.519	20860.852	20835.257	20877.869	20894.489	21042.436
MAPD%		0.8185	0.9355	0.8272	0.7866	0.0802

**Table 5:** Calculated energies (${cm^{-1}}$) for K${}_{2}$ (${X^1\Sigma _g^+}$) molecule (${\gamma =0.7975, \ \beta =0.6393}$).

${\nu }$	RKR^67^	IQSO^67^	ITP^67^	Eq. (53)	Eq. (50)
0	46.094	46.083	46.110	46.114	46.134
1	137.839	137.776	137.912	137.852	137.906
2	228.927	228.755	229.107	228.92	229.002
3	319.354	319.022	319.695	319.318	319.421
4	409.116	408.575	409.672	409.045	409.161
5	498.209	497.416	499.037	498.099	498.223
6	586.628	585.544	587.788	586.481	586.605
7	674.369	672.959	675.922	674.188	674.307
8	761.427	759.660	763.437	761.219	761.327
9	847.797	845.649	850.331	847.575	847.665
10	933.474	930.925	936.602	933.253	933.319
11	1018.451	1015.487	1022.248	1018.253	1018.291
12	1102.724	1099.337	1107.266	1102.573	1102.577
13	1186.286	1182.474	1191.654	1186.214	1186.177
14	1269.131	1264.898	1275.411	1269.173	1269.091
15	1351.252	1346.609	1358.532	1351.449	1351.318
16	1432.642	1427.606	1441.017	1433.043	1432.856
17	1513.294	1507.891	1522.863	1513.952	1513.706
MAPD%		0.2221	0.2994	0.0201	0.0194

**Table 6:** Calculated energies (${cm^{-1}}$) for CS (${X^1\Sigma ^+}$) (${\gamma =0.9222, \ \beta =0.7264}$), AsS (${X^2\prod }$) (${\gamma =0.9452, \ \beta =0.7328}$) and AsP (${X^1\Sigma ^+}$) (${\gamma =0.9267, \ \beta =0.7269}$) molecules.

	CS (${X^1\Sigma ^+}$)			AsS (${X^2\prod }$)			AsP (${X^1\Sigma ^+}$)		
${\nu }$	RKR^61^	Eq. (53)	Eq. (50)	RKR^60^	Eq. (53)	Eq. (50)	RKR^59^	Eq. (53)	Eq. (50)
0	640.9	640.8	641.2	283.47	283.76	284.26	301.51	301.40	301.72
1	1913.1	1912.6	1913.7	847.47	847.15	848.64	901.57	900.64	901.59
2	3172.3	3171.1	3172.9	1407.53	1405.98	1408.44	1497.67	1495.09	1496.65
3	4418.6	4416.2	4418.6	1963.65	1960.24	1963.66	2089.81	2084.76	2086.91
4	5652.0	5647.9	5651.0	2515.83	2509.95	2514.29			
5	6872.5	6866.3	6869.9	3064.07	3055.09	3060.34			
6	8080.1	8071.3	8075.5	3608.37	3595.66	3601.80			
7	9274.7	9262.9	9267.5						
MAPD%		0.0667	0.0357		0.1860	0.1207		0.1384	0.0694

**Table 7:** Calculated energies (${cm^{-1}}$) for SrO (${X^1\Sigma ^+}$) (${\gamma =0.7723, \ \beta =0.6363}$), YO (${X^2\Sigma ^+}$) (${\gamma =0.9247, \ \beta =0.7302}$) and ScO (${X^2\Sigma ^+}$) (${\gamma =0.9555, \ \beta =0.7561}$) molecules.

${\nu }$	SrO (${X^1\Sigma ^+}$)			YO (${X^2\Sigma ^+}$)			ScO (${X^2\Sigma ^+}$)		
	RKR^57^	Eq. (53)	Eq. (50)	RKR^58^	Eq. (53)	Eq. (50)	RKR^58^	Eq. (53)	Eq. (50)
0	325.76	326.03	323.25	429.7	429.71	429.81	481.4	481.4	481.2
1	971.33	974.04	965.73	1284.9	1284.71	1284.97	1437.9	1438.1	1437.7
2	1608.98	1616.55	1602.77	2134.1	2133.70	2134.11	2386.0	2386.7	2386.
3	2238.71	2253.57	2234.36	2977.6	2976.67	2977.22	3325.7	3327.2	3326.1
4	2860.52	2885.07	2860.51	3815.1	3813.61	3814.28	4257.0	4259.4	4258.1
5	3474.41	3511.07	3481.19	4646.8	4644.51	4645.29			
6	4080.38	4131.54	4096.42						
7	4678.43	4746.49	4706.18						
8	5268.56	5355.90	5310.47						
MAPD%		0.8639	0.4338		0.0260	0.0163		0.0305	0.0170

**Table 8:** Calculated energies (${cm^{-1}}$) for SiP (${X^2\prod }$) (${\gamma =0.7986,\ \beta =0.6341}$), SiN (${X^2\Sigma ^+}$) (${\gamma =0.7726,\ \beta =0.6137}$) and SiF${^+}$ (${X^1\Sigma ^+}$) (${\gamma =0.9296,\ \beta =0.7346}$) molecules.

${\nu }$	SiP (${X^2\prod }$)			SiN (${X^2\Sigma ^+}$)			SiF${^+}$ (${X^1\Sigma ^+}$)				
	RKR^56^	Eq. (53)	Eq. (50)	RKR^55^	Eq. (53)	Eq. (50)	RKR^54^	IGPT^31^	IRM^31^	Eq. (53)	Eq. (50)
0	306.74	307.60	308.72	574.1	573.5	576.42	523.95	523.91	523.89	523.89	523.91
1	917.75	917.24	920.53	1712.5	1707.4	1716.1	1564.43	1563.92	1564.39	1564.40	1564.40
2	1524.08	1520.82	1526.21	2838.0	2823.9	2838.1	2595.02	2593.57	2594.99	2595.01	2595.06
3	2125.74	2118.32	2125.76	3950.5	3923.0	3942.4	3615.72	3612.85	3615.70	3615.72	3615.76
4	2722.72	2709.74	2719.15	5050.1	5004.6	5028.9	4626.53	4621.78	4626.49	4626.51	4626.51
5	3315.02	3295.08	3306.4	6136.8	6068.6	6097.7	5627.44	5620.33	5627.34	5627.38	5627.32
6	3902.65	3874.33	3887.5								
7	4485.59	4447.49	4462.44								
MAPD%		0.4441	0.2979		0.6028	0.3130		0.0674	0.0031	0.3468	0.0021

## Discussion

In this part, the obtained results are applied to a selection of DMs with widespread uses in optical and molecular physics. First, the potential function curves for the chosen DMs are initially generated using the IRMP. The molecular parameters used in this study are presented in Table 1, which are collected from the literature^51–61^. In Figs. (1, 2, 3), potential function curves generated by the IRMP are displayed alongside the experimental RKR points for the considered DMs. These Figs. show that the generated IRMP curves closely correspond to the observed RKR data points^51–61^. We evaluate the average absolute deviations (AAD) from the RKR experimental data in order to demonstrate the effectiveness of the IRMP.

A prominent goodness-of-fit metric for evaluating the reliability of an empirical potential energy model is the AAD from the dissociation energy, which is defined as^62^.

58 $$\begin{aligned} \text {AAD}=\frac{100}{N D_e}\sum _r \Bigg |V_{RKR}(r)-V(r)\Bigg |, \end{aligned}$$

where ${V_{RKR}(r)}$ is the RKR potential and *N* is the number of experimental data points. Our AAD values for the chosen DMs are shown in Table 2. According to the Lippincott criterion,^62^ the AAD of the potential model must be less than 1${\%}$ of the dissociation energy in order to fit the RKR potential curve. Thus, a better model is indicated by the smaller value of the AAD.

As revealed by Table 2, the IRMP is a perfect model for simulating the RKR potential since the computed AAD outcomes for all of the considered DMs are less than 1${\%}$ of the dissociation energies. Further potential models for the K${}_{2}$(${X^1\Sigma _g^+}$) molecule that have AAD results are the Morse, Modified Morse, and Hulbert-Hirschfelder potentials^52^. Our AAD value is 0.6999${\%}$, whereas the AAD results for the Morse and Hulbert-Hirschfelder potentials are 2.395${\%}$, and 0.681${\%}$ respectively. Consequently, both the IRMP and Hulbert-Hirschfelder potential are superior to the Morse potential for simulating the RKR data of the K${}_{2}$(${X^1\Sigma _g^+}$) molecule.

In order to verify the reliability of the expressions generated for the IRMP using the GFNU technique, the pure vibrational energy levels of different DMs are computed in three-dimensional space (${D=3}$). Comparisons between the calculated energies and the experimental RKR data as well as earlier investigations are provided in Tables 3, 4, 5, 6, 7, 8. To further support the veracity of our findings, we also examine the mean absolute percentage deviation (MAPD) of the IRMP from the RKR experimental points. The MAPD is expressed as^50^:

59 $$\begin{aligned} {\text{MAPD}}=\frac{100}{N}\sum _{\nu} \Bigg |1-\frac{E_{\nu J}}{E_{RKR}}\Bigg |, \end{aligned}$$

where ${E_{RKR}}$ are the experimental RKR energies and ${E_{nJ}}$ are the computed energies using the IRMP. The vibrational energies of the selected DMs are calculated using Eqs. (50) and (53) in both the fractional and ordinary instances respectively. The results in Tables 3, 4, 5, 6, 7, 8 clearly show that the vibrational energies estimated using the IRMP are in close agreement with the RKR experimental data. Also for all of the chosen DMs, the calculated MAPD demonstrates that are within 1% of the allowed error from the experimental RKR values.

The vibrational energies of the ScI (${B^1\prod }$) molecule are displayed in Table 3, along with comparisons to the findings of Refs.^63–65^. Diaf et al. employed the path integrals formalism to compute the vibrational energies of the ScI (${B^1\prod }$) molecule with the q-deformed Scarf potential in Ref.^63^. While the modified forms of the generalised Mobius square and hyperbolical-type potentials were used in Refs.^64,65^. The findings of these comparisons show that they coincide with the other potential models^63–65^. The vibrational energies for the N${}_{2}$(${X^1\Sigma _g^+}$) molecule are listed in Table 4 compared to the observed RKR data and the outcomes of Refs.^52, 66^. The authors in Ref.^66^ employed the deformed hyperbolic barrier potential to calculate the energy levels of the N${}_{2}$(${X^1\Sigma _g^+}$) molecule. Whereas the authors of Ref.^52^ used the Morse and deformed modified Rosen-Morse (DMRM) potentials.

Table 4 illustrates that our findings agree better with the RKR data than those computed using the other potential models^52, 66^. Furthermore, our MAPD values are the smallest in both the ordinary and fractional cases. As a result, our IRMP estimates for modelling the N${}_{2}$(${X^1\Sigma _g^+}$) molecule are more accurate than the other works^52, 66^. The vibrational energies for the K${}_{2}$(${X^1\Sigma _g^+}$) molecule are reported in Table 5. When comparing our results with those of Eyube et al.^67^ for the K${}_{2}$(${X^1\Sigma _g^+}$) molecule, it becomes clear that our results from the IRMP are more precise for fitting the RKR data for the K${}_{2}$(${X^1\Sigma _g^+}$) molecule than those from the improved q-deformed Scarf oscillator (IQSO) and the ITP. The vibrational energies of the CS(${X^1\Sigma ^+}$), AsS(${X^2\prod }$) and AsP(${X^1\Sigma ^+}$) molecules are listed in Table 6. As illustrated in Table 6, our outcomes coincide with the RKR data. In Table 7, the computed values for the SrO(${X^1\Sigma ^+}$), YO(${X^2\Sigma ^+}$) and ScO(${X^2\Sigma ^+}$) molecules with the observed RKR values are presented. As can be seen in Table 7, the calculated and observed outcomes are in close agreement. The vibrational energies of the SiP(${X^2\prod }$) and SiN(${X^2\Sigma ^+}$) are listed in Table 8 molecules with the RKR experimental values. It appears that the estimated results and the RKR data agree well. In Table 8, we also provide a comparison of the computed vibrational energies for the SiF${^+}$(${X^1\Sigma ^+}$) molecule with the outcomes of Ref.^31^ and observed values. Yanar^31^ calculated the vibrational energies for the SiF${^+}$(${X^1\Sigma ^+}$) molecule using the IRMP as well as the improved generalized Pöschl-Teller (IGPT) potential . It is clear that the current findings for the SiF${^+}$(${X^1\Sigma ^+}$) molecule are in good accord with those of Ref.^31^. As illustrated in Tables 3, 4, 5, 6, 7, 8, the influence of incorporating fractional parameters on the vibrational energies for the molecules studied in this work is crucial for modelling the experimental RKR data. Consequently, our results can be investigated to examine various molecules in future studies.

## Conclusion

In this paper, the GFD is utilized for the first time to investigate the bound state solutions of the *D*-dimensional SE using the IRMP. Based on the GFNU, the analytical forms for the energy eigenvalues and wave functions of the IRMP are derived as a function of the fractional parameters in the *D*-dimensional space by employing the Pekeris-type approximation to the centrifugal term. The present results are applied to a number of DMs that have extensive applications in different physical domains. With the help of the molecular parameters, the potential energy curves are generated in terms of IRMP for the selected DMs. For the chosen DMs, the AAD of the IRMP from the observed RKR data is presented. According to our estimated AAD, the IRMP can successfully fit the experimental RKR data of several DMs. To validate the mechanism used in this research, the pure vibrational energies for different DMs are calculated in both ordinary (${\gamma =\beta =1}$) and fractional (${\gamma \ne 1, \beta \ne 1}$) cases in three-dimensional space (${D=3}$). It is found that the current computed pure vibrational energy values are preferable to those from earlier works and are in full harmony with the experimental data. It is further shown that the pure vibrational energies of different DMs computed in the existence of fractional parameters fit the observed RKR data better than those computed in the ordinary case. This leads one to the conclusion that fractional order significantly affects the vibrational energy levels of DMs. The MAPD from the observed RKR data points is assessed to further substantiate the accuracy of our findings. According to the assessed MAPD, our values are accurate to within a 1% error margin of the experimental RKR values. Therefore, the current findings indicate that the IRMP is a precise model for estimating the observed RKR data for all of the DMs considered in this investigation.

## Data Availability

All data generated or analysed during this study are available upon reasonable request from the corresponding author.
